# Augmenting Anticancer Immunity Through Combined Targeting of Angiogenic and PD-1/PD-L1 Pathways: Challenges and Opportunities

**DOI:** 10.3389/fimmu.2020.598877

**Published:** 2020-11-05

**Authors:** Stephen P. Hack, Andrew X. Zhu, Yulei Wang

**Affiliations:** ^1^ Product Development (Oncology), Genentech, Inc., South San Francisco, CA, United States; ^2^ Massachusetts General Hospital Cancer Center and Harvard Medical School, Boston, MA, United States; ^3^ Jiahui International Cancer Center, Jiahui Health, Shanghai, China

**Keywords:** programmed death ligand 1 (PD-L1), vascular endothelial growth factor (VEGF), angiogenesis, checkpoint inhibitor, tumor microenvironment, programmed death-1 (PD-1)

## Abstract

Cancer immunotherapy (CIT) with antibodies targeting the programmed cell death 1 protein (PD-1)/programmed cell death 1 ligand 1 (PD-L1) axis have changed the standard of care in multiple cancers. However, durable antitumor responses have been observed in only a minority of patients, indicating the presence of other inhibitory mechanisms that act to restrain anticancer immunity. Therefore, new therapeutic strategies targeted against other immune suppressive mechanisms are needed to enhance anticancer immunity and maximize the clinical benefit of CIT in patients who are resistant to immune checkpoint inhibition. Preclinical and clinical studies have identified abnormalities in the tumor microenvironment (TME) that can negatively impact the efficacy of PD-1/PD-L1 blockade. Angiogenic factors such as vascular endothelial growth factor (VEGF) drive immunosuppression in the TME by inducing vascular abnormalities, suppressing antigen presentation and immune effector cells, or augmenting the immune suppressive activity of regulatory T cells, myeloid-derived suppressor cells, and tumor-associated macrophages. In turn, immunosuppressive cells can drive angiogenesis, thereby creating a vicious cycle of suppressed antitumor immunity. VEGF-mediated immune suppression in the TME and its negative impact on the efficacy of CIT provide a therapeutic rationale to combine PD-1/PD-L1 antibodies with anti-VEGF drugs in order to normalize the TME. A multitude of clinical trials have been initiated to evaluate combinations of a PD-1/PD-L1 antibody with an anti-VEGF in a variety of cancers. Recently, the positive results from five Phase III studies in non-small cell lung cancer (adenocarcinoma), renal cell carcinoma, and hepatocellular carcinoma have shown that combinations of PD-1/PD-L1 antibodies and anti-VEGF agents significantly improved clinical outcomes compared with respective standards of care. Such combinations have been approved by health authorities and are now standard treatment options for renal cell carcinoma, non-small cell lung cancer, and hepatocellular carcinoma. A plethora of other randomized studies of similar combinations are currently ongoing. Here, we discuss the principle mechanisms of VEGF-mediated immunosuppression studied in preclinical models or as part of translational clinical studies. We also discuss data from recently reported randomized clinical trials. Finally, we discuss how these concepts and approaches can be further incorporated into clinical practice to improve immunotherapy outcomes for patients with cancer.

## Introduction

Over the past decade, cancer immunotherapy (CIT) has dramatically changed the treatment landscape of cancer. This major therapeutic advance was made possible in large part by pioneering preclinical and clinical research focused on immune modulation using antibodies that block immune regulatory checkpoints (1–5).

Immune checkpoints such as cytotoxic T-lymphocyte-associated protein 4 (CTLA-4), programed cell death protein 1 (PD-1), and programed cell death 1 ligand 1 (PD-L1) act to negatively regulate T-cell–mediated immune responses that play a critical role in allowing cancer cells to evade the immune destruction. Immune checkpoint inhibitors (ICIs) are monoclonal antibodies directed against either the PD-1/PD-L1 axis or CTLA-4. ICIs attenuate inhibitory T-cell activation signals, thereby permitting tumor-reactive T cells to overcome regulatory mechanisms in order to mount an effective antitumor response (6). At the time of writing, a total of 10 PD-1/PD-L1 monoclonal antibodies are approved by regulatory authorities either as monotherapy or in combination across different lines of treatment for 19 different types of cancer, including a tissue-agnostic indication (**Supplemental Table 1**). As of September 2019, approximately 3,000 trials involving drugs targeting the PD-1/PD-L1 axis are ongoing across a range of tumors types, with 76% of them evaluating combination regimens (7). Given this rapid pace of clinical development, it is anticipated that more PD-1/PD-L1–based treatments will change the standard of care in many more cancer types.

A hallmark of drugs that inhibit the PD-1/PD-L1 axis is the induction of deep and durable antitumor responses that can translate into a survival benefit in patients with a variety of tumor histologies. Anti–PD-1 treatment has resulted in marked improvements in 5-year survival for patients with advanced melanoma, lung cancer, and renal cancer over previous standards of care (8). However, long-term responses are restricted to a minority of patients and an estimated 87% of patients’ cancers for which PD-1/PD-L1 are indicated will fail to respond (9). Most patients experiencing resistance to PD-1/PD-L1 inhibition either never respond to treatment (primary resistance) or relapse after a period of response (acquired resistance). Furthermore, some tumor types such as pancreatic, microsatellite stable (MSS) colorectal, biliary tract, and prostate cancers appear intrinsically resistant to PD-1/PD-L1 axis blockade (10). A major reason accounting for both primary or acquired resistance is the ability of tumors to exploit alternate immune-suppressive mechanisms, thereby circumventing checkpoint blockade (11). Collectively, the tumor microenvironment (TME), tumor immunogenicity, antigen presentation, as well as oncologic signal transduction pathways all play important roles in response and resistance to immune checkpoint blockade (10).

As the molecular mechanisms underlying resistance to ICI are unearthed, actionable therapeutic strategies to prevent or abrogate them are being developed to improve clinical outcomes for patients. Tumor mutation burden (TMB)—which reflects the abundance of immunogenic neoantigens that are identified as foreign by cytotoxic T cells—and expression of inhibitory immune checkpoints such as PD-L1 have been widely studied as biomarkers of response to checkpoint inhibitors (CPIs). However, neither of these markers can fully explain the lack of response to checkpoint blockade observed in the majority of patients (10, 12–16). It is therefore likely that other immunosuppressive mechanisms act to restrain anticancer immunity. Abnormalities within the TME are strongly associated with repressed anticancer immunity, which profoundly impacts the effectiveness of immunotherapy (11, 17–19). Thus, therapeutic reprogramming of specific immune components of the TME with combination treatments, such as immunosuppressive cell types, may overcome TME-induced resistance to checkpoint blockade, thereby enhancing or reinvigorating anticancer immunity (17, 20). ICIs in combination with treatment modalities such as chemotherapy, targeted agents and CTLA-4 antibodies have been successfully developed and further studies are ongoing to evaluate other combination approaches including radiation and immune modulators [recently reviewed by Murciano-Goroff et al. (21)]. Each of these combination partners is thought to modulate anticancer immunity *via* direct and indirect mechanisms (21, 22). PD-1 inhibitors along with anti-CTLA-4 antibodies have received FDA approvals for a range of cancers and trials involving other inhibitory checkpoints, such as LAG-3 and TIM-3 are ongoing (23–25). Conventional cytotoxic chemotherapies promote anticancer immunity through the release of tumor-associated antigens and/or depletion of immunosuppressive cells (26, 27). Chemotherapy regimens in tandem with ICIs have been extensively studied and have become treatment options for NSCLC, triple-negative breast cancer and urothelial carcinoma (28–31). Similar to chemotherapy, radiation treatment can augment the anticancer immune response through the release of tumor antigens and modulation of the TME (21, 32). Studies of ICIs with radiation are ongoing in a variety of cancers (21, 33). ICIs combined agents targeting components of the MAP-kinase pathway have also been evaluated (21, 34–36). Within the TME, vascular endothelial growth factor (VEGF)–driven angiogenesis is a key driver of tumor-associated immunosuppression. VEGF-mediated immunosuppression has been extensively studied in a variety of preclinical and clinical studies, which collectively have highlighted the mechanisms underpinning combined immune checkpoint blockade and VEGF inhibition in patients with cancer.

In this comprehensive review, we focus on the mechanisms underpinning VEGF-mediated immunosuppression and how these can be therapeutically abrogated by combined VEGF and PD-(L)1 blockade in patients with cancer to augment antitumor immunity. These mechanistic concepts and clinical approaches are very relevant and timely given that combinations of PD-(L)1 inhibitors and antiangiogenic agents are either currently approved or are close to approval for the treatment of a variety of malignancies. We also highlight the opportunities and challenges associated with dual targeting of VEGF and PD-(L)1 pathways.

## Intersection Between Anticancer Immunity and Angiogenesis

**Figure 1:**
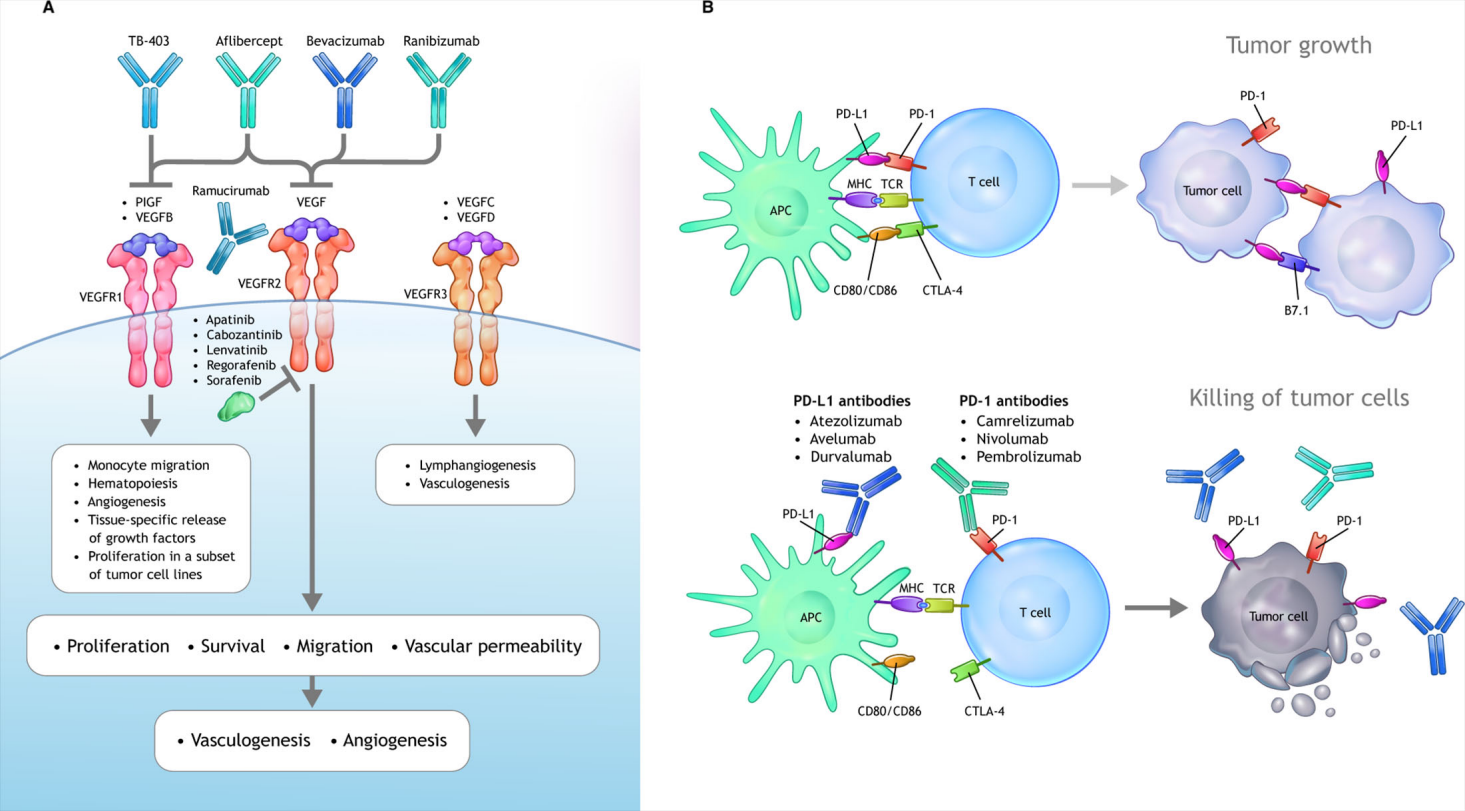
VEGF and PD-1/PD-L1 signaling axes. **(A)** VEGF ligands include VEGF-A, VEGF-B, VEGF-C, VEGF-D, and PlGF, which interact with a combination of various VEGFRs. Canonical VEGF signaling through VEGF-R1/R2 (with R2 being the dominant signaling receptor) regulates the activities of several kinases and ultimately guides cell proliferation, migration, survival, and vascular permeability during vasculogenesis and angiogenesis. Multiple inhibitors block VEGFA-induced signaling. Bevacizumab and ranibizumab bind VEGFA. The soluble chimeric receptor aflibercept binds VEGFA, PlGF, and VEGFB. The VEGFR2-specific monoclonal antibody ramucirumab prevents VEGFR2-dependent signaling. Numerous small molecule TKIs block VEGFR signaling. **(B)** Activated T cells express PD-1, which engages with its specific ligand (PD-L1 or PD-L2) to dampen activation. PD-1 axis blockade through the administration of an anti–PD-1 or anti–PD-L1 antibody prevents this inhibitory interaction and unleashes antitumoral T lymphocyte activity by promoting increased T-cell activation and proliferation, by enhancing their effector functions. APC, antigen-presenting cells; CTL, cytotoxic T lymphocytes; DC, dendritic cell; iDCs, immature dendritic cells; IL, interleukin; iMC, immature myeloid cells; M1, classical macrophages; M2, alternative macrophages; matDCs, mature dendritic cells; MDSC, myeloid-derived suppressor cell; PD-1, programmed cell death 1 protein; PD-L1, programed cell death ligand 1; PlGF, placental growth factor; TAM, tumor associated macrophages; TFG-β, transforming growth factor β; TKI, tyrosine kinase inhibitor; Treg, tumor-associated macrophages; VEGF, vascular endothelial growth factor; VEGFR, vascular endothelial growth factor receptor.

Angiogenesis and immune evasion are interdependent processes that often occur in parallel and are considered hallmarks of cancer **(** 37, 38 **)**. Both are physiological mechanisms that can be hijacked in cancer, facilitating tumor development and progression **(** 38 **)** (**Figure 1**).

### The Cancer-Immunity Cycle

Cancer immunity was characterized by Chen and Mellman as a seven-step, self-propagating, cyclical, multistep process, referred to as the cancer-immunity cycle (CIC) (39). In order for effective antitumor immunity to occur, a series of stepwise events that enable T-cell–mediated tumor cell killing is necessary. The seven steps of the CIC can be grouped into 3 distinct phases (40):

1. Recruitment and activation of immune effector cells (steps 1–3);
2. Trafficking and infiltration of T cells into tumors (steps 4 and 5);
3. Recognition and killing of cancer cells (steps 6 and 7).

In steps 1 through 3 of the CIC, tumor antigens (including neoantigens) liberated from tumor cells are taken up and processed by dendritic cells (DCs) and are then presented to T cells that results in the priming and activation of T-cells. In step 4, activated effector T cells enter the circulation, are trafficked to the tumor and then infiltrate the tumor bed (step 5), where they attach to and destroy cancer cells (steps 6 and 7). The killing of malignant cells leads to the additional release of tumor-derived antigens and the restarting of the CIC. Tumors are able to co-opt mechanisms to evade immune surveillance by obstructing one or more steps in the CIC, thus rendering tumors safe from immune destruction.

Knowledge of the mechanisms underpinning anticancer immunity has led to the development of classification systems characterizing the TME that help identify patients who are more likely to respond to immunotherapy and also serves as a framework to inform rational combination treatments (12, 41, 42). Current classifications are primarily defined according to the composition of the immune infiltrate and the character of the inflammatory response (43). Histologically, tumors can be broadly categorized as either inﬂamed (“hot”) or noninﬂamed (“cold”) (42). Most data support the idea that patients with hot or inflamed tumors, which harbor markers of preexisting functional antitumor T-cell immunity [e.g., interferon (IFN)-γ signaling, high PD-L1 expression, high prevalence of tumor-infiltrating lymphocytes (TILs), or genomic instability], tend to respond relatively well to PD-1/PD-L1 inhibition (12, 41, 42). Other tumor immune phenotypes with deficits in antitumoral immunity such as those with immune-excluded (immune cells present only in the periphery) and immune-desert (with limited or no infiltration of immune cells in to the tumor)—are not as likely to respond to CPIs, suggesting the existence of other vital immune-suppressive mechanisms in either the tumor or the TME (12, 42). The immune phenotypes described above can be present to varying degrees within a given tumor type and among different cancers (12).

### VEGF Immunomodulation

Angiogenesis, defined as new blood vessel formation from the preexisting vasculature, is a complex, multistep process that under physiological conditions is tightly regulated by a plethora of proangiogenic and antiangiogenic factors (44). However, in malignant settings, proliferating tumors tend to activate angiogenesis by shifting the balance of proangiogenic and antiangiogenic mediators toward a proangiogenic outcome (referred to as the “angiogenic switch”).

Of all the molecules known to regulate angiogenesis, VEGF and its receptors (VEGFRs) have received the most attention due to VEGF’s key role in regulating physiological and pathological angiogenesis (45). VEGF belongs to a family of growth factors that includes VEGFs A to D and placental growth factor. VEGF (VEGF-A) binds to both R1 and R2 VEGFR subtypes as well as the neuropilin 1 receptors (46) (**Figure 1**). The binding of VEGF to VEGFR2 is the primary signaling event in blood endothelial cells triggering angiogenesis (46). VEGF binding to VEGFR initiates various intracellular signaling pathways that regulate processes such as vascular permeability and endothelial cell survival, migration, and proliferation (47). The role played by VEGFR1 is unclear. VEGFR1 binds VEGF-A with a higher affinity than VEGFR2 (approximately 10 times) but possesses weak kinase activity; it is hypothesized that VEGFR1 may act to sequester VEGF-A away from VEGFR2 (48).

Antiangiogenic drugs can be classified according to three mechanisms of action: monoclonal antibodies that bind and deplete the VEGF ligand, monoclonal antibodies that bind to the VEGFR, and tyrosine kinase inhibitors (TKIs) that block the intracellular domain of the VEGFR. The role of VEGF in oncogenesis and signaling mechanisms and the development of anti-VEGF therapeutics have been reviewed in detail elsewhere (47, 49). A summary of US Food and Drug Administration (FDA)-approved anti-VEGF agents and their indications is provided in **Supplemental Table 2**.

**Figure 2:**
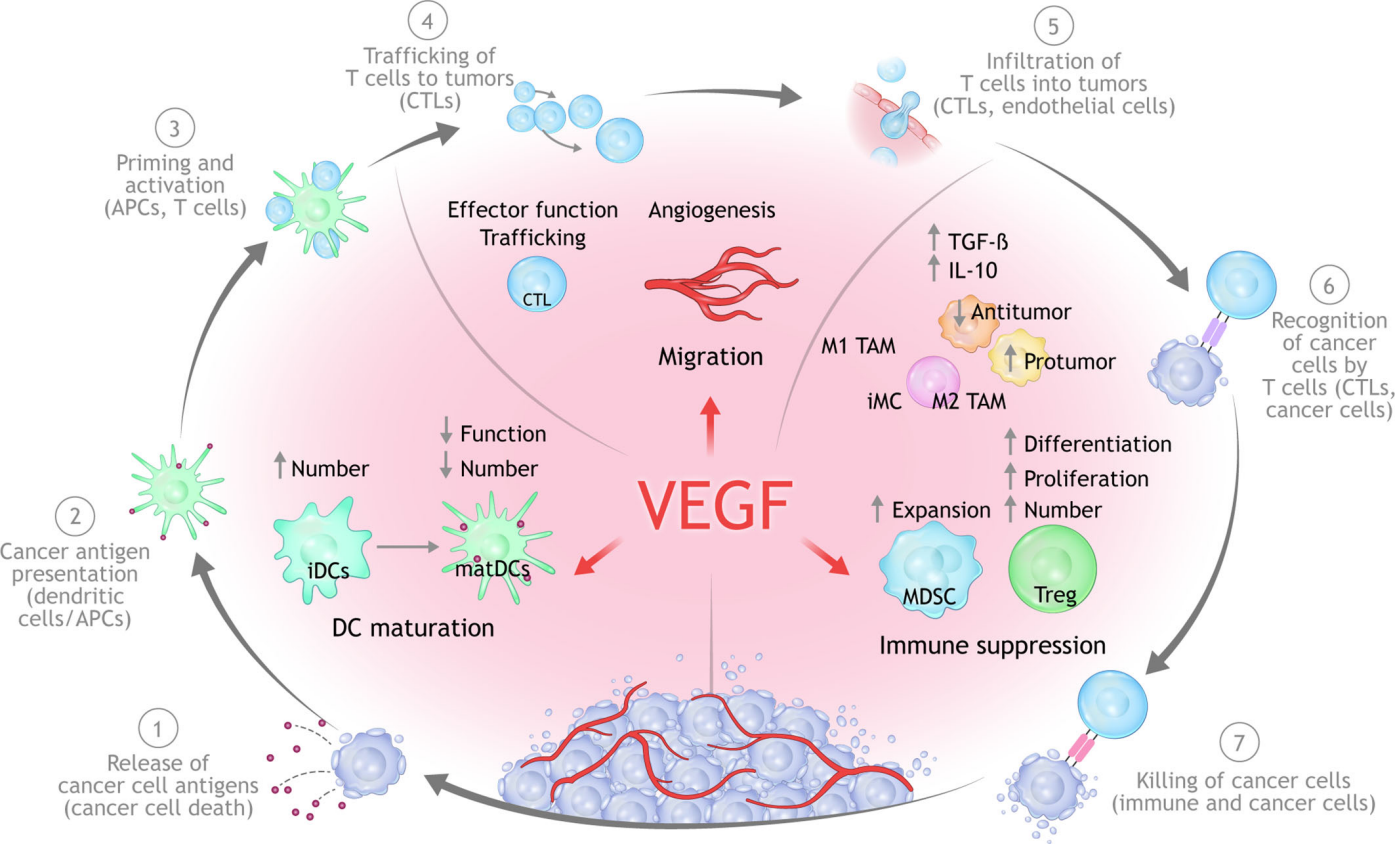
Mechanisms of VEGF-mediated immunosuppression in the TME. Beyond its ability to mediate immune suppression *via* an abnormal tumor vasculature, increased VEGF levels can lead to immune suppression *via* inhibition of DC maturation, reduction of T-cell tumor infiltration, and promotion of inhibitory cell types in the TME. APC, antigen-presenting cells; CTLA, cytotoxic T lymphocyte associated; DC, dendritic cell; MHC, major histocompatibility complex; PD-1, programmed cell death 1 protein; PD-L1, programed cell death ligand 1; PlGF, placental growth factor; TME, tumor microenvironment; TCR, T-cell receptor; VEGF, vascular endothelial growth factor; VEGFR, vascular endothelial growth factor receptor.

In addition to vascular regulation, emerging and evolving data have implicated VEGF as an important mediator of immunosuppression within the TME (13). VEGF is able to drive a range of immunosuppressive mechanisms impacting the ability to mount an effective anticancer immune response (38, 39) (**Figure 2**).

Overproduction of VEGF in the TME can drive suppress antitumor immunity either directly or indirectly *via* four principle mechanisms (13, 38, 50):

1. Inhibition of DC maturation and antigen presentation;
2. Inhibition of cytotoxic T-cell proliferation, trafficking, and infiltration;
3. Promotion of an aberrant tumor vasculature;
4. Recruitment and proliferation of immunosuppressive cell types, e.g., MDSCs, regulatory T cells (Tregs), and pro-tumor M2-like tumor-associated macrophages.

Here, we describe the mechanistic interplay between VEGF and the CIC (51). Key preclinical studies are summarized in **Table 1**.

**Table 1:** Selected preclinical studies.

Checkpoint Inhibitor	Antiangiogenic Therapy	Tumor Model	Key Results^a^	Reference
**Anti–PD-1 mAb (clone RMP-014)**	DC101 (anti-VEGFR2 mAb)	Hepatocellular carcinoma	* Anticancer activity ↑ * Animal survival ↑ * CD8^+^ T-cell infiltration and activation ↑ * CD4^+^-mediated vessel normalization * PD-1/PD-L1 expression ↑ with anti-VEGFR2 block (mediated by IFN-γ) * M2 → M1 shift in TAMs * Treg and CCR2^+^ infiltration ↓	Shigeta et al. (52)
**Anti–PD-1 mAb; (clone RMP1-14)**	Lenvatinib (TKI targeting VEGFR 1-3, FGFR 1-4, PDGFRα, KIT, and RET)	Hepatocellular carcinoma	* Anticancer activity ↑ * Response rate ↑ * CD8^+^ T cells ↑ * Macrophages and monocytes ↓	Kimura et al. (53)
**Anti–PD-1 mAb (clone RMP-014)**	DC101 (anti-VEGFR2 mAb)	Colon cancer	* Anticancer activity ↑ * Animal survival ↑ * TOX-dependent T-cell exhaustion induced by VEGF-A * Reinvigoration of exhausted T cells	Kim et al. (54)
**Anti–PD-L1 mAb (clone 6E11)**	Anti-VEGF mAb (B20-4.1.1)	SCLC	* Animal PFS and OS ↑ * Rescue of exhausted T-cell phenotype	Meder et al. (55)
**Anti–PD-1 mAb (clone RMPI-14)**	DC101	Colon cancer	* Angiogenesis ↓ * T-cell infiltration ↑ * Cytokine expression ↑	Yasuda et al. (56)
**Anti–PD-1 mAb (clone RMP1-14)**	Sunitinib (VEGFR TKI)	Colon cancer	* PD-1^+^CD8^+^ T cells ↓ * Anticancer activity ↑	Voron et al. (57)
**Anti–PD-L1 mAb (clone 10F.9G2)**	DC101	* Pancreatic cancer * Breast cancer * Glioblastoma	* IFNγ-expressing CD8+ and IFNγ-expressing CD4+ T cells ↑ * Anti–PD-L1 enhanced antiangiogenic efficacy in pNET and BC, but not GBM * PD-L1 expression on relapsing tumor cells ↑ * Vessel normalization ↑ by PD-L1 blockade and formation of HEVs ↑ *via* LTβR	Allen et al. (58)
**Anti–PD-1 mAb (clone 29F.1A12**	Axitinib	* Lung * Colon	* Mast cells ↓ * TAMs ↓ * T-cell depletion ↓ axitinib antitumor activity and survival * Axitinib induced ↓ checkpoint expression on CD8^+^ T cells * Axitinib + anti–PD-1 ↑ animal survival	Läubli et al. (59)

↑ indicates increased cell numbers or an improvement in outcome compared with those observed with control treatments. ↓ indicates decreased cell numbers or a decrease in the outcome measured compared with control treatments.

CCR2^+^, chemokine (C-C motif) receptor 2–positive monocyte; HEVs, high endothelial venules; LTβR, lymphotoxin-β receptor; mAb, monoclonal antibody; MDSCs, myeloid-derived suppressor cells; NA, not applicable; PD-1, programmed cell death protein 1; PD-L1, programmed cell death 1 ligand 1; sVEGFR, soluble VEGF receptor; TAMs, tumor-associated macrophages; TOX, thymocyte selection-associated high mobility group box protein; TKI, tyrosine kinase inhibitor; Treg, regulatory T cell; VEGFR, VEGF receptor.

a Comparisons are between combined therapy and monotherapy or control treatments (see references for details).

#### Dendritic Cell Maturation (CIC Steps 1, 2, and 3)

Steps 1 through 3 of the CIC refer to the activation and recruitment of immune cells (39, 40). Step 1 encompasses the release and capture of tumor neoantigens by DCs. DCs are antigen-presenting cells that play a critical role in T-cell priming and the activation of anticancer T cells (steps 2 and 3 of the CIC).

T-cell priming and activation of cytotoxic T cells is reliant upon the ability of mature DCs to capture and present tumor antigens to T cells in the lymph nodes (39, 60). However, tumor-associated DCs exist in an immature state and are often unable to properly contribute to initiating a functional anticancer immune response. The ability to inhibit DC maturation, which can result in deficient tumor-antigen presentation and thus in potential immune evasion by tumors, was one of the first-described immunosuppressive functions of VEGF (61). Mature DCs are characterized by increased expression of MHC I and II and other costimulatory molecules on the cell surface that are required for T-cell activation, all of which are under the regulation of the nuclear factor-κB pathway (51). However, in cancers harboring elevated VEGF levels, DC maturation can be impeded through nuclear factor-κB pathway inhibition as a result of VEGF-VEGFR1 binding on DCs. This lack of DC maturation can prevent the upregulation of MHC and other molecules, ultimately resulting in impaired T-cell activation. VEGF, acting through VEGFR2, has also been shown to inhibit the ability of mature DCs to stimulate T cells (62). VEGFR1 and VEGFRs may have differential roles in regulating DC differentiation where VEGFR1 is the principle mediator of VEGF-induced inhibition of DC maturation (63). Neuropilin 1 has also been implicated in VEGF-mediated inhibition of DC maturation (64). Furthermore, by upregulating PD-L1 on DCs, VEGF can further suppress DC function, resulting in suppressed T-cell function and/or expansion (65). High levels of VEGF expression in human cancers have been linked with defective DC function and a reduction in mature DCs, especially in advanced-stage tumors (38, 50).

Data from *in vitro* studies show that VEGF was able to inhibit the differentiation of monocytes into DCs which could be restored with treatment with bevacizumab or sorafenib, a multi–tyrosine kinase VEGFR2 inhibitor (66). Relatedly, bevacizumab treatment has been shown to increase the number of mature DCs in peripheral blood of cancer patients (67).

A recent study reported that DCs are regulated by PD-L1, and through blocking PD-L1, T-cell priming was augmented by the activation of the PD-L1/B7.1 signaling axis (68). In the same study, patients with either renal cell carcinoma (RCC) or non-small cell lung cancer (NSCLC) harboring a high DC signature before treatment were more prone to respond to PD-L1 inhibition with atezolizumab.

In summary, studies show that DCs are regulated by both the PD-L1 and VEGF signaling axes. Multiple studies demonstrate that VEGF can drive immunosuppression partly through inhibition of DC maturation and can facilitate immune evasion as a result of attenuated T-cell activation and priming which. Taken together, these findings suggest that combined inhibition of PD-1/PD-L1 and VEGF could result in enhanced activation and recruitment of T cells *via* regulation of DC function and maturation.

#### T-Cell Proliferation, Trafficking, and Infiltration (CIC Steps 4 and 5)

The trafficking of primed and activated T cells from the lymph node to the tumor bed are highlighted in Steps 4 and 5 of the CIC. Anticancer immunity is imparted by both tumor-infiltrating immune cells residing in tissue as well as in the blood (13). Successful blockade of PD-1/PD-L1 is reliant on effective trafﬁcking of tumor-targeted T cells from lymph nodes, through the blood stream, and into the tumor (69). As a result, resistance to PD-1/PD-L1 inhibition is often linked with inadequate T-cell infiltration into the tumor prior to treatment (12, 41, 42). To effectively infiltrate the tumor and integrate into the TME, immune cells must be able to enter the tumor vasculature, attach to the endothelium, and then migrate across the vessel wall (39). The trafficking of primed and activated T cells from the lymph node into circulation and then to the tumor is dependent on a series of steps that includes T-cell rolling and adhesion to the vascular endothelium (69, 70).

VEGF plays a critical role in this process by stimulating abnormal vasculature formation in the tumor, which can negatively impact T-cell migration from lymph nodes into the tumor bed (40, 45, 51). VEGF, as well as other immunosuppressive factors, can attenuate the expression of adhesion molecules (e.g., intracellular adhesion molecule 1, vascular adhesion molecule 1, CD34) on the vascular endothelium of the tumor. Reduced expression of adhesion molecules acts to impair the ability of immune cells to adhere to and migrate across the vessel wall, thereby preventing their entry into the tumor (71). Other studies have suggested that VEGF exposure can lead to the abnormal clustering of adhesion molecules on endothelial cells, resulting in reduced T-cell adhesion (72).

Endothelial cells can express a range of molecules that serve to create an impermeable barrier to certain immune cells (13). One such molecule is FAS antigen ligand. In combination with prostaglandin E2 and IL-10, FAS antigen ligand acquired the ability to induce apoptosis of CD8^+^ T cells but not Tregs (73). Pharmacologic blockade of VEGF-A induced a marked increase in the influx of tumor-rejecting CD8^+^ T cells over Tregs that was dependent on attenuation of FAS antigen ligand expression and led to CD8-dependent tumor growth suppression (73). Studies in cancer patients have shown links between tumor angiogenesis, tumor vascular dysfunction, or elevated VEGF-A levels and diminished tumor T-cell inﬁltration (74).

T-cell exhaustion, characterized by the expression of negative immune checkpoints such as PD-1 receptors that result in a progressive loss of function, is an important mechanism of anticancer immune evasion. Studies in mouse models have shown that increased VEGF-A in the TME can enhance the expression of PD-1—as well as other receptors involved in T-cell exhaustion—on CD8^+^ T cells, which could be prevented by anti VEGF treatment (57).

In summary, many of the immunosuppressive effects of VEGF are mediated by abnormalities in the tumor vasculature that are driven by VEGF, which can subsequently prevent effective T-cell infiltration and promote tumor immune evasion. Further, pharmacologic blockade of VEGF promotes the infiltration of cytotoxic T cells into tumors.

#### Vascular Normalization

Aberrant angiogenesis as well as physical compression leads to abnormal vessels and impaired blood perfusion in tumors (45). Abnormal vessels mediate immune escape and can reduce the efficacy of immunotherapy by hampering the delivery of drugs, oxygen, and effector T cells. Abnormal tumor blood vessels are prone to hypoxia and acidosis within the TME, which mediates suppressed anticancer immunity through several mechanisms (13, 45). As a result, alleviating vascular dysfunction—a process referred to as “vascular normalization”—could both improve the delivery and efficacy of anticancer treatments and overcome TME immunosuppression (75). Studies in mice have demonstrated that modulation or normalization of tumor vasculature can result in increased T-cell recruitment and inﬁltration into tumors (76, 77). In turn, vascular function can also be regulated by immune cells, as shown by a recent study in experimental breast tumors models in which effector CD4^+^ T cells, introduced by either adoptive transfer or dual PD-1/CTLA4 blockade, were found to both normalize blood vessels and attenuate hypoxia (78). Relatedly, in breast and colon tumor models, anti-PD-1 or anti-CTLA-4 treatment boosted vessel perfusion through the promotion of CD8^+^ T-cell accumulation and IFN-γ production, suggesting that improved vessel perfusion was contingent on upregulated T-cell immunity induced by checkpoint blockade (79). These data indicate that both vascular and T-cell function are mutually regulated processes in cancer.

#### Recruitment and Proliferation of Immunosuppressive Cells (CIC Steps 6 and 7)

Steps 6 and 7 of the CIC rely on a permissive TME in which the balance of effector T cells and immune suppressive cells permits the recognition and killing of tumor cells (51). VEGF-mediated immunosuppression is caused by both negative effects on immune effectors and the augmentation of immune suppressive cells such as Tregs, MDSCs, and TAMs with pro-tumor phenotypes (17, 80). In addition to downregulating anticancer immunity, suppressive cells can also drive angiogenesis, thereby creating a vicious cycle of immunosuppression (80). Reprogramming of the TME from immune suppressive to immune permissive may be possible by blocking VEGF-induced expansion of MDSCs, Tregs and other immune suppressive cells which would lead to activation of antitumor immunity (17, 39).

##### Myeloid Cells

Myeloid cells include macrophages, neutrophils, and MDSCs. MDSCs play a critical role in regulating anticancer immunity in the TME as well as resistance to PD-1/PD-L1 antibodies. MDSCs are a diverse population of myeloid cells existing in various states of differentiation that display potent immune suppressive functions (81, 82). MDSCs also potentiate angiogenesis *via* different mechanisms (83).

MDSCs facilitate tumor progression *via* two principle mechanisms: (1) immune suppression by perturbation of immune effector function (T cells and natural killer cells) and the induction of Tregs; (2) promotion of angiogenesis (77). MDSCs in the TME are able to suppress the proliferation of tumor-specific T cells and promote Treg development or differentiation, leading to suppressed T-cell immunity. The binding of VEGF to VEGFR on MDSCs activates signaling *via* signal transducer and activator of transcription 3, resulting in their expansion (83). Although the pro-tumoral effects of MSDCs have been ascribed to immune-related parameters, non-immune mechanisms such as promotion of angiogenesis also foster cancer progression and metastasis (84). A recent study reported that PD-1 signaling regulates the lineage fate and functionality of myeloid cells in mice. Specifically, selective PD-1 ablation in myeloid cells was found to be more effective at inhibiting tumor growth than global PD-1 deletion in T cells (85, 86). In addition, targeted ablation of PD-1 on myeloid cells was shown to induce an increase of T-effector memory cells with improved functionality which allowed for effective antitumor protection despite functional PD-1 expression in T cells.

In a RCC mouse model, bevacizumab was shown to reduce the number of MDSCs (87). Sunitinib (a VEGFR TKI) increases TILs and reduced MDSCs in human RCC (88). In a murine model of RCC, sunitinib markedly reduced the infiltration of MDSCs into tumors, as well as reduced MDSCs in the peripheral blood of patients with RCC (89). MDSCs are also implicated in resistance to VEGF blockade in both mouse models as well as patients with cancer (89–91). In a syngeneic murine model of hepatocellular carcinoma (HCC), antibody targeting of tumor-infiltrating MDSCs improved the anticancer activity of sorafenib (90).

Collectively, these data indicate that myeloid cell function is orchestrated by both VEGF and PD-1 pathways, highlighting the rationale for therapeutic PD-1/PD-L1 plus VEGF inhibition in cancers in which myeloid-driven immunosuppression blunts an effective anticancer immune response.

##### Regulatory T-Cells

Regulatory T-cells (Tregs) are potent mediators of TME immunosuppression (92) and are regulated by several tumor-secreted factors, including VEGF (93, 94). VEGF has been shown to trigger Treg recruitment and proliferation (93). For example, VEGF blockade can lead to decreased numbers of Tregs in the TME both in CRC mouse models and patients with CRC treated with combination of bevacizumab and chemotherapy (95). Further, the hypoxic conditions that result from VEGF-mediated abnormalities in the tumor vasculature can also induce secretion of chemokine CCL28 from tumor cells that leads to Treg recruitment, and accumulation of immunosuppressive M2 tumor-associated macrophages. Through these actions, excessive VEGF creates an immune suppressive TME that downregulates tumor-specific T-cell function, thereby facilitating tumor immune evasion (13, 17). Anti-VEGF treatment has been shown to reduce tumor-related Tregs in patients with RCC (96).

##### Other Immunosuppressive Cell Types

MDSCs and Tregs act in concert with other immunosuppressive cell types in the TME that are regulated by VEGF and contribute suppressed antitumor immunity (17). For example, in a murine HCC model dual anti–PD-1/VEGFR2 treatment significantly inhibited primary tumor growth and (52) and successfully reprogrammed the TME through increased CD8^+^ T-cell infiltration and activation, shifting the M1:M2 ratio of TAMs, and reducing Treg and chemokine receptor 2 infiltration in HCC tissue. In addition, combination treatment induced durable vessel fortification. Similar immunomodulatory effects have been reported with lenvatinib, an anti-VEGFR TKI, combined with an anti–PD-1 antibody in murine HCC models (53, 97).

### Data From Clinical Biomarker Studies

Accumulating clinical biomarker data from studies in RCC and HCC have offered mechanistic insights into how VEGF blockade can overcome ICI resistance.

The combination of atezolizumab and bevacizumab was evaluated in two clinical studies of patients with advanced RCC in which immune markers were correlated with clinical efficacy to investigate the mechanisms underpinning PD-L1/VEGF inhibition (98, 99).

In Phase I study, 10 patients with RCC were treated initially with bevacizumab to evaluate the effects of bevacizumab on the TME, followed by combination therapy with atezolizumab (98). Serial biopsies and blood draws were performed at baseline, following bevacizumab, and 4 to 6 weeks after commencing combination treatment. Treatment with bevacizumab alone resulted in upregulation of MHC I staining by immunohistochemistry (IHC). Interestingly, this response was coupled with other favorable effects in the TME such as increased CD8^+^ T-cell and macrophage infiltration, as well as increased gene signature expression related to T-helper and CD8+ T-effector cells, natural killer cells, and chemokines.

In addition to favorable immune-related changes, bevacizumab alone or bevacizumab plus atezolizumab also induced changes in vascular parameters such as decreases in expression of neovasculature-related genes, staining of vessel-lining endothelial cell marker CD31 in the tumor, and microvascular density (98). Reduced microvascular density was associated with enhanced CD8+ T-cell tumor staining by IHC, suggesting increased T-cell infiltration. Of note, patients treated with atezolizumab and bevacizumab had more CD8^+^ T-cell tumor infiltration than those treated with bevacizumab alone.

Subsequently, a randomized Phase II trial was undertaken to evaluate the efficacy of atezolizumab with or without bevacizumab compared with sunitinib as a first-line treatment of clear-cell RCC (99). This study included biomarker analysis (high vs. low) to study three biological axes: angiogenesis, preexisting immunity, and myeloid immune suppression. The combination of atezolizumab plus bevacizumab had a marked PFS benefit over sunitinib or atezolizumab monotherapy in patients with tumors harboring elevated expression of a myeloid inflammation signature and T-effector signature, whereas sunitinib had greater efficacy than the combination in patients with tumors with high levels of angiogenesis (99). These exploratory data suggest that myeloid-induced immune suppression might act to restrain antitumor immune responses induced by atezolizumab and that the addition of bevacizumab could act to circumvent this restraint (50).

More recently, a genomic correlative study from a randomized Phase Ib cohort evaluating atezolizumab alone or in combination with bevacizumab in unresectable HCC was presented (100). Similar to the RCC analysis described above (99), this study evaluated immunological biomarker subpopulations defined according to gene signatures (characterized as high vs. low relative to the median). The progression-free survival (PFS) benefit of combination treatment compared with atezolizumab alone was particularly marked in patients with HCC who had high expression of the following biomarkers: VEGFR2 gene (*KDR)*, myeloid, Tregs, and triggering receptor expressed on myeloid cells-1 (TREM-1) (**Table 2**). These observations are consistent with mechanisms implicated in preclinical studies in murine HCC models, as well as data showing that VEGF/VEGFR2 blockade can inhibit Treg and MDSC accumulation tumors or blood in human cancers (52, 63, 95, 101). Although these findings require validation, they provide direct evidence that myeloid- and/or Treg-mediated immunosuppression play an important role in mediating resistance to PD-L1 blockade and that these mechanisms can be therapeutically abrogated with anti-VEGF therapy.

**Table 2:** PFS benefit with atezolizumab plus bevacizumab compared vs. atezolizumab alone in subpopulations of patients by HCC exploratory biomarkers.

Biomarker Subpopulation		Atezolizumab + Bevacizumab vs. Atezolizumab PFS, HR (95% CI)	*N* per Group(combo, mono)
**VEGFR2**	VEGFR2^high^	0.36 (0.16–0.81)	21, 25
	VEGFR2^low^	0.88 (0.4–1.9)	23, 22
**Tregs**	Treg^high^	0.35 (0.15–0.82)	21, 25
	Treg^low^	0.82 (0.39–1.7)	23, 22
**Myeloid**	Myeloid^high^	0.43 (0.19–0.95)	22, 24
	Myeloid^low^	0.80 (0.37–1.7)	22, 23
**TREM**	TREM^high^	0.43 (0.10–0.94)	24, 22
	TREM^low^	0.77 (0.36–1.6)	20, 25

HCC, hepatocellular carcinoma; PFS, progression-free survival; Treg, regulatory T cells; TREM, triggering receptor expressed on myeloid cells-1.

It remains to be seen whether the therapeutically relevant immune suppressive mechanisms described in this section are broadly applicable across cancer types or vary depending on tumor histology and tissue-specific immune regulation (102).

## Anti-VEGF as Immunotherapy: Evidence From Clinical Trials

The intriguing preclinical and translational clinical studies highlighting the immunomodulatory effects of VEGF blockade described in the previous section have resulted in a myriad of clinical trials testing the combination of PD-1/PD-L1 antibodies with anti-VEGF drugs (**Tables 3** and **4**) (7). Positive Phase III studies have led to recent approvals by the FDA for dual PD-1/PD-L1 and anti-VEGF combinations in RCC (pembrolizumab plus axitinib, and avelumab plus axitinib), endometrial carcinoma (pembrolizumab plus lenvatinib), non-squamous NSCLC (atezolizumab, bevacizumab and chemotherapy), and HCC, suggesting a potential broad clinical utility of this combination strategy (104, 105, 107, 109–111). Given the number of clinical studies, the following section focuses primarily on randomized trials for which results are available.

**Table 3:** Ongoing randomized Phase II or Phase III studies of PD-1/PD-L1 antibodies combined with VEGF inhibitors.

Anti-VEGF	PD-1/PD-L1	Other Drugs/Interventions	Tumor Type	Study Phase	n	Primary Endpoint(s)	NCT ID (study name)
**Bevacizumab**	Atezolizumab	Paclitaxel + carboplatin	Recurrent OC, FTC, or PPC	III	1300	PFS/OS	NCT03038100 (IMagyn050)
**Bevacizumab**	Atezolizumab	Paclitaxel or pegylated liposomal doxorubicin	Recurrent OC	III	664	PFS/OS	NCT03353831
**Bevacizumab**	Atezolizumab	Carboplatin + gemcitabine, carboplatin + paclitaxel or carboplatin + pegylated liposomal doxorubicin	OC	III	600	PFS	NCT02891824 (ATALANTE/ENGOT OV29)
**Bevacizumab**	Atezolizumab	Pegylated liposomal doxorubicin hydrochloride	Recurrent OC, FTC, or PPC	II/III	488	PFS/OS	NCT02839707
**Bevacizumab**	Atezolizumab	Aspirin	Recurrent platinum-resistant OC, FTC or PPC	II	160	PFS at 6 months	NCT02659384 (EORTC-1508)
**Bevacizumab**	Durvalumab	Carbo/tax Olaparib	1L OC	III	1056	PFS in BRCA non-mut	NCT03737643 (DUO-O)
**Bevacizumab**	Atezolizumab	FOLFOX	1L dMMR mCRC	III	347	PFS	NCT02997228 (COMMIT)
**Bevacizumab**	Atezolizumab	FOLFOXIRI	1L mCRC	II	201	PFS	NCT03721653 (AtezoTRIBE)
**Bevacizumab**	Nivolumab	N/A	Recurrent GBM	II	90	OS at 12 months	NCT03452579
**Bevacizumab**	Nivolumab	FOLFOX	1L mCRC	II/III	180	PFS	NCT03414983 (CheckMate 9X8)
**Bevacizumab**	Atezolizumab	carboplatin and pemetrexed	1L NSCLC (non-squamous)	II	117	PFS	NCT03786692
**Bevacizumab**	Nivolumab	Carboplatin/paclitaxel	1L NSCLC (non-squamous)	III	530	PFS	NCT03117049 (TASUKI-52)
**Bevacizumab**	Pembrolizumab	Chemotherapy	1L cervical cancer	III	600	PFS/OS	NCT03635567 (KEYNOTE-826)
**Bevacizumab**	Atezolizumab	Chemotherapy	1L cervical cancer	III	404	OS	NCT03556839 (BEATcc)
**Bevacizumab**	Atezolizumab	Carboplatin/pemetrexed	1L pleural mesothelioma	III	320	PFS/OS	NCT03762018 (BEAT-meso)
**Bevacizumab**	Atezolizumab	N/A	Adjuvant HCC	III	662	RFS	NCT04102098 (IMbrave 050)
**Bevacizumab**	Durvalumab	N/A	Adjuvant HCC	III	888	RFS	NCT03847428 (EMERALD-2)
**Bevacizumab**	Durvalumab	TACE	Intermediate-stage HCC	III	600	PFS	NCT03778957 (EMERALD-1)
**Bevacizumab**	Durvalumab	N/A	1L HCC	II	433	Safety	NCT02519348
**Cabozantinib**	Atezolizumab	N/A	1L HCC	III	740	PFS/OS	NCT03755791 (COSMIC-312)
**Apatinib**	SHR-1210	N/A	IL HCC	III	510	PFS/OS	NCT03764293
**Lenvatinib**	Pembrolizumab	N/A	Recurrent endometrial cancer	III	780	PFS/OS	NCT03517449 (KEYNOTE-775)
**Lenvatinib**	Pembrolizumab	N/A	1L advanced endometrial cancer	III	720	PFS/OS	NCT03884101 (LEAP-001)
**Lenvatinib**	Pembrolizumab	N/A	1L HCC	III	750	PFS/OS	NCT03713593 (LEAP 002)
**Lenvatinib**	Pembrolizumab	N/A	1L RCC	III	1069	PFS	NCT02811861 (CLEAR)
**Cabozantinib**	Nivolumab	N/A	1L RCC	III	638	PFS	NCT03141177 (CheckMate 9ER)
**Cabozantinib**	Nivolumab	Ipilimumab	1L RCC	III	1046	OS	NCT03793166 (PDIGREE)
**Cabozantinib**	Nivolumab	Ipilimumab	1L RCC	III	676	PFS	NCT03937219 (COSMIC-313)

CRC, colorectal carcinoma; dMMR, mismatch repair deficient; ER, estrogen receptor; FTC, fallopian tube cancer; GBM, glioblastoma; HCC, hepatocellular carcinoma; HER2, human epidermal growth factor receptor 2; m, metastatic; MSS, microsatellite stable; N/A, not applicable; NSCLC, non-small cell lung cancer; OC, ovarian cancer; OS, overall survival; PD-1, programmed cell death protein 1; PD-L1, programmed cell death 1 ligand 1; PFS, progression-free survival; pMMR, mismatch repair proficient; PPC, primary peritoneal cancer; RCC, renal cell carcinoma; TACE, transarterial chemoembolization; UC, urothelial carcinoma.

Studies included in **Table 1** are not included.

**Table 4:** Completed randomized studies of PD-1/PD-L1 antibodies combined with VEGF inhibitors in solid tumors.

Experimental Arm(s)	Control Arm	Tumor	Phase	Primary Endpoint(s)	OS	PFS	ORR (vs. control)	NCT ID (study name)	Reference
**Bevacizumab + Atezolizumab**	Sunitinib	1L RCC	III	PFS (PD-L1+); OS (ITT)	ITT Population OS HR: 0.93; (0.76–1.14; *P* = 0.4751)^a^	PD-L1 HR, 0.74 (95% CI, 0.57–0.96), *P =* 0.02 ITT HR, 0.83 (95% CI: 0.70–0.97)	PD-L1+ 43% vs. 35% ITT 37% vs. 33%	NCT02420821 (IMmotion151)	Rini (103),. *Lancet* 393, 2404–2415.
**Bevacizumab + Atezolizumab + chemo**	Chemo + bevacizumab	1L NSCLC	III	PFS in ITT-WT; PFS in Teff-high WT; OS in ITT-WT	ITT-WT HR, 0.78 (95% CI, 0.64–0.96; *P =* 0.02)	ITT-WT HR, 0.62 (95% CI, 0.52–0.74; *P <* 0.001)	ITT-WT ORR: 64% vs. 48%	NCT02366143 (IMpower150)	Socinski et al. *N Engl J Med*, (104)
**Bevacizumab + Atezolizumab**	Sunitinib	1L RCC	II	PFS in ITT and PD-L1+	NR	ITT HR, 1.00 (95% CI, 0.69–1.45) PD-L1+ HR, 0.64 (95% CI, 0.38–1.08)	ITT 32% vs. 29% PD-L1+ 46% vs. 27%	NCT01984242 (IMmotion150)	McDermott et al. *Nat Med* (99)
**Axitinib + Pembrolizumab**	Sunitinib	1L RCC	III	PFS/OS	HR 0.53; (95% CI, 0.38–0.74; *P* < 0.0001)	HR: 0.69; (95% CI, 0.57–0.84; *P* = 0.0001)	59% vs. 36%; *P* < 0.0001)	NCT02853331 (Keynote 426)	Motzer, (105). *N Engl J Med* 380, 1103–1115.
**Axitinib + Avelumab**	Sunitinib	1L RCC	III	PFS/OS (PD-L1+)	0.82 (95% CI, 0.53– 1.28; *P =* 0.38)	0.61 (95% CI, 0.47– 0.79; *P <* 0.001)	ORR: 55% vs. 26%	NCT02684006 (Javelin RENAL)	Motzer et al. (105). *N Engl J Med* 380, 1103–1115.
**Bevacizumab + Atezolizumab**	Atezolizumab	1L HCC	Ib	PFS (Arm F)	NR	PFS HR: 0.55; (80% CI, 0.40–0.74; *P =* 0.011)	ORR: 20% vs. 17%	NCT01633970	Lee et al. (106) *Lancet Oncol* 21, 808–820
**Bevacizumab + Atezolizumab**	Sorafenib	1L HCC	III	PFS/OS	OS HR: 0.58 (0.42– 0.79; *P <* 0.001)	PFS HR: 0.59; (95% CI, 0.47– 0.76; *P <* 0.001)	ORR: 27% vs. 12% (*P <* 0.001)	NCT03434379 (IMbrave150)	Finn et al. (107). *N Engl J Med* 382, 1894–1905.
**Bevacizumab + Atezolizumab + capecitabine**	Capecitabine + bevacizumab	Chemo refractory mCRC	II	PFS	HR 0.94 (0.56–1.56; *P =* 0.398)	HR 0.73 (95% CI, 0.49–1.07; *P =* 0.051)	ORR: 9% vs. 4%	NCT02873195 (BACCI)	Mettu et al. (108) (ESMO)

HCC, hepatocellular carcinoma; HR, hazard ratio; INV, investigator-assessed; ITT, intention-to-treat; NA, not available; NCT ID, ClinicalTrials.gov identifier; NR, not yet reached; NSCLC, non-small cell lung cancer; OS, overall survival; PD-L1, programmed death-ligand 1; PFS, progression-free survival; RCC, renal cell carcinoma; Teff, T-effector gene signature (PD-L1, CXCL9, and interferon-γ); WT, wild-type.

^a^Results did not cross the prespecified significance boundary of α = 0.0009 at the first interim analysis.

### Renal Cell Carcinoma

Clear-cell RCCs, which make up approximately 70% of RCC cases, are associated with a hyperangiogenic state that is brought on by VEGF overproduction resulting from inactivation of the von Hippel–Lindau tumor-suppressor gene (112). As a result, multiple VEGF-directed therapies are approved for the treatment of RCC (**Supplemental Table 2**).

As well as being highly angiogenic, RCC is also immunogenic, as evidenced by responsiveness to PD-1/PD-L1 axis blockade (99, 113). These clinical findings, coupled with emerging data regarding the immunomodulatory actions of anti-VEGF drugs, led to multiple studies combining anti-VEGF agents with PD-1/PD-L1 antibodies (114). The combination of ICIs and antiangiogenics has been tested most extensively in patients with advanced RCC.

Early clinical studies in patients with RCC demonstrated encouraging antitumor activity of these combination regimens along with a manageable safety profile (94, 115, 116). However, some combinations involving VEGF TKIs were associated with excessive toxicity that precluded further development and highlighted the need for careful selection of the antiangiogenic agent (117). To date, five Phase III studies have been initiated to evaluate various combinations of VEGF or VEGFR inhibitors plus either PD‐1 or PD‐L1 antibodies in patients with advanced RCC, of which three have been published (103, 105, 109). Based on the results of JAVELIN 101 and KEYNOTE-426, combination treatment with either pembrolizumab or avelumab plus axitinib is now considered a standard of care in frontline advanced RCC (118, 119).

#### IMmotion151

IMmotion151 was a randomized Phase III study comparing atezolizumab plus bevacizumab vs. sunitinib in patients with advanced RCC (103). Co-primary endpoints were investigator-assessed PFS in the PD-L1^+^ population and overall survival (OS) in the intention-to-treat (ITT) population. A total of 915 patients were randomized to receive either atezolizumab plus bevacizumab or sunitinib. Bevacizumab plus atezolizumab significantly improved PFS compared with sunitinib in patients with PD‐L1^+^ tumors (HR, 0.74; *P* = .02) and in the ITT population (HR, 0.83). In the ITT population, OS did not cross the significance boundary at the interim analysis (HR, 0.93).

#### Javelin 101

Javelin 101 was a randomized Phase III study comparing avelumab plus axitinib vs. sunitinib in patients with advanced RCC (105). A total of 886 patients were randomized to either avelumab plus axitinib or sunitinib. The combination of axitinib plus avelumab significantly improved PFS compared with sunitinib in patients with PD‐L1^+^ tumors (HR, 0.61; *P* <.001) and in the ITT population (HR, 0.69; *P* <.001). PD‐L1^+^ patients had objective response rates (ORRs) of 55.2% vs. 25.5% in favor of axitinib plus avelumab. OS was immature at the time of data cutoff. In the ITT population, axitinib plus avelumab treatment resulted in an ORR of 51% compared with 26% with sunitinib.

#### KEYNOTE-426

KEYNOTE-426 was a randomized Phase III study comparing pembrolizumab plus axitinib to sunitinib in patients with advanced clear-cell RCC (109). A total of 861 patients were randomized to receive either pembrolizumab plus axitinib or sunitinib. The combination of axitinib plus pembrolizumab significantly improved both OS (HR, 0.53; *P* < 0.0001) and PFS (HR, 0.69; *P* < 0.001) compared with sunitinib in the ITT population. Notably, KEYNOTE‐426 was the first of the combination studies to demonstrate an OS benefit over sunitinib in RCC. ORR, a secondary endpoint, was also significantly improved with axitinib plus pembrolizumab compared with sunitinib (59.3% vs. 35.7%; *P* < 0.0001).

### Colorectal Cancer

The clinical benefit of ICIs in metastatic colorectal cancer (mCRC) is confined to the 4% to 5% of patients with tumors with deficient DNA mismatch repair pathways (dMMR) or high microsatellite instability (MSI-H) (120, 121). Conversely, PD-(L)1 inhibitors do not show clinically relevant activity in proficient DNA mismatch repair pathways (pMMR) or MSS mCRC (120, 122). The marked response to anti–PD-1 therapy in dMMR/MSI-H mCRC can be explained by high levels of tumor mutation burden (123–125). However, mutation burden alone cannot explain the lack of response to anti–PD-1 treatment in MSS/pMMR mCRC (15, 126). Factors other than mutational burden might therefore account for the lack of response to checkpoint blockade in MSS/pMMR mCRC.

The differential response to CPI in dMMR/MSI-H and MSS/pMMR mCRC is likely due in part to differences in the TME that impact the tumor’s ability to mount an effective anticancer immune response (127). VEGF is believed to play a fundamental role in shaping the immune-suppressive TME in MSS CRC. Recent data from a series of *in vitro*, *in vivo*, and *ex vivo* studies demonstrated that severe T-cell exhaustion driven by VEGF-A was highly prominent in MSS CRC tumors compared with MSI-H CRC tumors (54). T-cell exhaustion in MSS CRC tumors was characterized by diminished CD8^+^ T-cell infiltration at the invasive margin and tumor body, upregulated expression of exhaustion markers such as PD-L1, and reduced IFN-γ release. The frequency of a wound-healing gene signature characterized by the elevated expression of angiogenic genes was found in 81% of MSS CRC tumors compared with 40% of MSI-H tumors. Furthermore, VEGF expression was markedly higher in MSS vs. MSI-H CRC tumors and VEGF was found to drive T-cell exhaustion as well as reduced T-cell functionality in MSS tumors. Together, these data give mechanistic insights into the role of VEGF-mediated suppression of T-cell immunity in CRC tumors and provide a rational framework to clinically evaluate co-targeting VEGF and PD-1/PD-L1 pathways.

#### dMMR/MSI-H Colorectal Cancer

MSI‐H/dMMR status is a biomarker associated with poor prognosis in mCRC and is predictive for response to immune CPIs (128). Phase II studies demonstrated durable responses of MSI‐H/dMMR tumors to PD-1 inhibitors (120, 128, 129). Pembrolizumab was recently shown in a Phase III study to significantly improve PFS vs. chemotherapy as first-line therapy for patients with MSI-H/dMMR mCRC (130).

Despite high levels of response to PD-1 blockade, not all patients with dMMR/MSI-H disease respond or subsequently develop resistance, potentially as a result of mechanisms similar to those observed in other cancers, including VEGF (129). The combination of atezolizumab and bevacizumab was studied in a cohort of 10 patients with heavily pretreated MSI-H mCRC and resulted in an ORR of 30% and a disease control rate of 90% (131).

The immunomodulatory role of VEGF in colon cancer was retrospectively studied in the NSABP C-08 study of adjuvant FOLFOX plus bevacizumab in stage II/III colon cancer (132). In the overall study population, bevacizumab did not significantly improve disease-free survival (HR, 0.89). However, in a *post hoc* analysis of patients harboring either dMMR or pMMR, bevacizumab was associated with improved survival compared with FOLFOX alone in the dMMR subgroup (133). By contrast, no survival benefit was seen in the pMMR subgroup. This result suggests that inhibition of VEGF alone, at least in some groups of patients with CRC and preexisting anticancer immunity, provides an immunostimulatory effect sufficient to augment the anticancer immune response and provides a rationale to combine bevacizumab with a CPI to amplify immunity (38). A Phase III study is ongoing to evaluate the combination of FOLFOX and bevacizumab with or without atezolizumab in first-line mCRC with dMMR (134).

#### MSS Colorectal Cancer

Unlike dMMR/MSI-H mCRC, patients with MSS mCRC (who account for around 95% of patients) anti–PD-(L)1 therapy has demonstrated limited or no clinical benefit (124, 135–138).

Treatment with chemotherapy and bevacizumab has been shown to induce positive immunological changes (e.g., increase in total lymphocytes, increase in CD4 and CD8 T cells) in the peripheral blood of patients with mCRC (139). However, these favorable changes were largely transient and had dissipated by cycle 6 of treatment. This suggests that amplifying these chemotherapy/anti-VEGF-induced immunomodulatory effects with a CPI could be beneficial.

In a Phase I study of 14 patients with refractory MSS CRC treated with atezolizumab plus bevacizumab, 1 patient (7%) had an objective response and 9 patients (64%) had stable disease (140). In a cohort of 23 patients with first-line mCRC, an ORR of 52% was reported, along with a median PFS of 14.1 months (95% CI, 8.7–17.1) and a median duration of response of 11.4 months (141). Interestingly, a single patient experienced a durable complete radiological response in a liver lesion.

These preliminary Phase I data prompted the initiation of a number of randomized studies evaluating the combination of PD-(L)1 antibodies and anti-VEGF drugs in mCRC. The BACCI study, a placebo-controlled randomized Phase II study, evaluated the addition of atezolizumab to bevacizumab and capecitabine in refractory mCRC (108). Approximately, 86% of randomized patients had MSS mCRC. In the overall study population (n = 133), atezolizumab plus capecitabine/bevacizumab significantly improved PFS compared with capecitabine/bevacizumab (median PFS, 3.3 vs. 4.4 months; HR, 0.73; *P =* 0.051). In patients with MSS tumors, the PFS benefit was more pronounced (HR 0.67). Response rate and OS were not significantly increased (108). Maintenance therapy with atezolizumab following induction treatment with FOLFOX plus bevacizumab in first-line mCRC was evaluated in the MODUL study (142). A total of 696 patients without B-Raf proto-oncogene, serine/threonine kinase (*BRAF*) mutations were randomized to either fluorouracil (5-FU)–bevacizumab plus atezolizumab or 5-FU–bevacizumab (143). The study did not meet its primary PFS endpoint (HR, 0.92; *P =* 0.48). In an updated analysis, the PFS outcome was unchanged and survival was not significantly increased (HR, 0.86; *P =* 0.28).

In an effort to improve the immune recognition of colorectal tumors, atezolizumab was combined with cobimetinib [a mitogen-activated protein kinase kinase (MEK) inhibitor] and bevacizumab in patients with previously treated mCRC in a Phase I trial (144). The rationale for pairing a PD-L1 antibody with a MEK inhibitor was the results of a preclinical study that showed that MEK pathway blockade augmented the antitumor activity of CPIs by increased T-cell infiltration into tumors and increased MHC-1 and PD-L1 expression (145, 146). The combination of atezolizumab, bevacizumab, and cobimetinib had an ORR of 8% in both patients with second-line mCRC and those with refractory mCRC; 92% of patients had MSS disease and 8% had unknown MSI status (144). PFS and OS appeared to be enhanced in patients harboring RAS mutations. Notably, atezolizumab combined with cobimetinib did not improve survival in refractory mCRC compared with standard-of-care regorafenib in a Phase III study (35). Because of the equivocal clinical benefit of the atezolizumab-bevacizumab-cobimetinib combination, further clinical development was halted (144).

The combination of regorafenib and nivolumab was studied in a Phase Ib trial in Japanese patients with refractory CRC or gastric cancer, 98% of whom had MSS disease (147). In 25 patients with heavily pretreated MSS CRC, ORR was 33%. Unlike in the GC cohort, in the limited number of patients with CRC no clear relationship between PD-L1 or TMB and efficacy outcomes was observed, and therefore, additional analysis is necessary to clarify the optimal patient population for this combination.

Taken together, the efficacy of combined PD-(L)1 antibodies plus anti-VEGF in MSS mCRC is inconclusive and may suggest that the combination could be efficacious in as-yet unidentified subgroups of patients. The data also point to the highly immunosuppressed nature of MSS colorectal tumors and the need for novel strategies to circumvent inherent immune resistance. A Phase II/III trial (CheckMate 9X8) is currently ongoing to evaluate nivolumab in combination with bevacizumab and FOLFOX (**Table 3**).

### Lung Cancer

NSCLC accounts for 80% to 85% of lung cancers, and adenocarcinoma and squamous cell carcinoma are the most common NSCLC histologic subtypes (148). CPIs have revolutionized the treatment of NSCLC, and most patients with newly diagnosed advanced NSCLC are indicated for treatment with PD-1 or PD-L1 antibodies, either as monotherapy or in combination (149, 150).

The IMpower150 study was designed to evaluate the clinical benefit of PD-L1 blockade with the immunomodulatory effects of chemotherapy and anti-VEGF (17, 28, 151–153). IMpower150 was a Phase III randomized trial comparing atezolizumab plus bevacizumab and chemotherapy (carboplatin and paclitaxel) to standard-of-care chemotherapy plus bevacizumab in patients with stage IV non-squamous NSCLC (104). A total of 1,202 patients were randomized to one of three arms to receive either:

- atezolizumab plus carboplatin-paclitaxel (ACP group)
- atezolizumab plus bevacizumab plus carboplatin-paclitaxel (ABCP group)
- bevacizumab plus carboplatin-paclitaxel (BCP group).

Patients treated with ABCP had improved PFS vs. patients receiving BCP therapy (HR, 0.62; P < .001) as well as improved OS (HR 0.78; P = .02). In addition, ORR (a secondary endpoint) was also increased in patients who received ABCP vs. BCP (64% vs. 48%, respectively). The clinical benefit of ABCP compared with BCP extended across key patient subgroups irrespective of PD-L1 expression levels, presence of baseline liver metastases (unstratified PFS HR, 0.42; unstratified OS HR, 0.54), and EGFR/ALK genetic alterations (unstratified PFS HR, 0.59; unstratified OS HR, 0.54).

**Figure 3:**
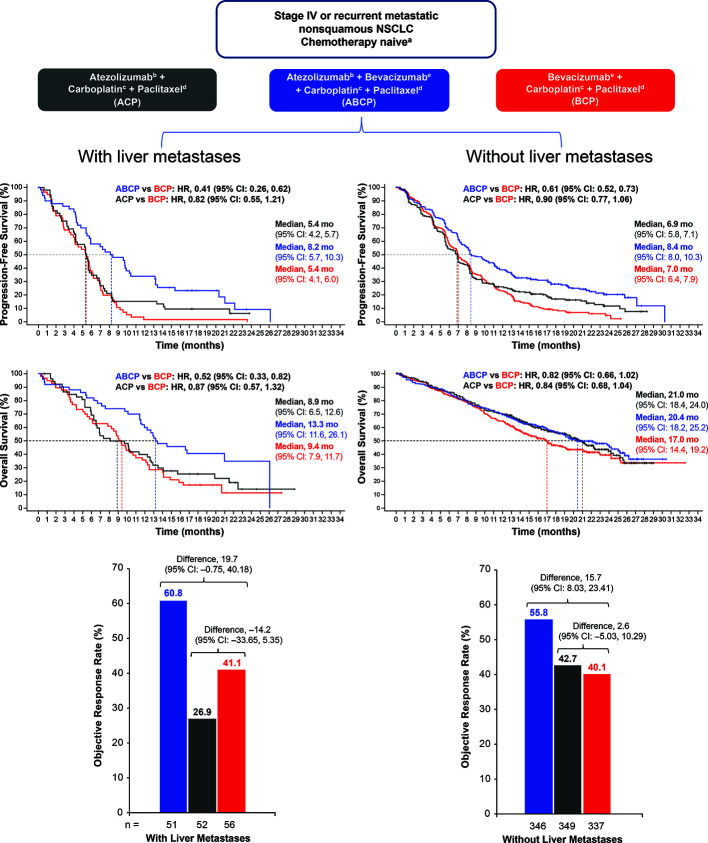
PFS, OS, and ORR in patients with NSCLC with or without baseline liver metastases. Kaplan-Meier estimates of PFS and OS in patients with or without liver metastases at baseline in the intention-to-treat population for the ABCP vs. BCP treatment comparison and the ACP vs. BCP treatment comparison. Adapted from Reck et al. (154). ABCP, atezolizumab plus bevacizumab plus carboplatin plus paclitaxel; ACP, atezolizumab plus carboplatin plus paclitaxel; BCP, bevacizumab plus carboplatin plus paclitaxel; CI, confidence interval; HR, hazard ratio; NSCLC, non-small cell lung cancer; ORR, objective response rate; OS, overall survival; PFS, progression-free survival.

In contrast to the OS benefit observed in patients treated with ABCP vs. BCP (HR, 0.76), no survival benefit (HR 0·85) was seen in those receiving ACP compared with the standard-of-care BCP regimen in the ITT population (154). Furthermore, whereas treatment with ABCP markedly improved PFS and OS relative to BCP in patients with activating EGFR mutations or liver metastases, the ACP regimen (without bevacizumab) did not show improved PFS or survival compared with BCP in these important clinical subgroups (154) (**Figure 3**). Relatedly, the lack of efficacy seen with the ACP regimen in IMpower150 in patients with EGFR mutations or baseline liver metastases was also observed in IMpower130, which evaluated atezolizumab in combination with carboplatin plus nab-paclitaxel chemotherapy (29). Together, these data indicate that bevacizumab, *via* restraint of angiogenesis and reversal of VEGF-driven immunosuppression in the TME, is needed in addition to atezolizumab and chemotherapy to unleash clinically effective anticancer immunity in patients with NSCLC harboring an *EGFR* mutation or liver metastases (50). Liver metastases are discussed further in a subsequent section.

On the basis of these data, carboplatin-paclitaxel in combination with bevacizumab plus atezolizumab is considered a standard-of-care first-line treatment for patients with non-squamous metastatic NSCLC (155). Additional randomized trials evaluating PD-(L)1–VEGF-chemotherapy combinations are currently ongoing in patients with NSCLC (**Table 3**).

### Gynecological Cancers

Gynecologic malignancies are among the most prevalent cancers affecting women worldwide. With the exception endometrial cancer, in advanced gynecologic cancers CPIs have demonstrated only limited antitumor activity, highlighting the need for combination strategies to bolster anticancer immunity in these tumors (156).

#### Endometrial Cancer

Up to 30% of endometrial cancers are MSI-H/dMMR and respond well to anti–PD-(L)1 inhibitors. However, response to PD-(L)1 blockade in MSS/pMMR endometrial tumors is modest, highlighting an unmet need for combination CIT regimens to augment anticancer immunity (157). Dual PD-1 and VEGF inhibition is one such combination that has been evaluated in advanced endometrial cancer.

The combination of pembrolizumab and lenvatinib in advanced primary or recurrent endometrial cancer, independent of MMR status, was studied in a single arm Phase II study (110, 111). In the final efficacy analysis, the ORR (by irRECIST) in 108 previo15usly treated patients was 38% at week 24 per investigator review, with a median PFS of 7.5 months (111). ORR was 64% in patients with MSI-H tumors (n = 11) and 36% in patients with MSS tumors (n = 94) (158). These encouraging preliminary data led to accelerated FDA approval of the combination of lenvatinib with pembrolizumab for the treatment of advanced endometrial cancer that is not MSI-H/dMMR and has progressed following prior therapy (157). The combination of lenvatinib and pembrolizumab is now under study in two ongoing Phase III trials (**Table 3**): lenvatinib with pembrolizumab vs. doxorubicin or weekly paclitaxel in advanced recurrent endometrial cancer (NCT03517449) and frontline lenvatinib with pembrolizumab vs. carboplatin and paclitaxel chemotherapy in advanced endometrial cancer (NCT03884101). There is also an ongoing Phase II single-group study evaluating bevacizumab and atezolizumab in recurrent endometrial cancer (NCT03526432).

#### Cervical Cancer

The standard treatment for recurrent or metastatic cervical cancer is a combination of chemotherapy and bevacizumab (159–162), but treatment options for recurrent disease are limited. Almost all cervical cancers are mediated by human papillomavirus infection which, when considered alongside relatively high mutation burden and expression of PD-L1, makes immunotherapy a potentially attractive treatment strategy (163, 164).

Despite having favorable immune biology, patients with cervical cancer have seen modest activity with single-agent CPIs (164, 165). The Phase II nonrandomized KEYNOTE-158 study evaluated pembrolizumab in 98 patients with recurrent or metastatic cervical cancer who had progressed on or were intolerant to at least one line of standard therapy and reported an ORR of 12% (166); all responses occurred in PD-L1–positive tumors. On the basis of these data, the FDA granted accelerated approval of pembrolizumab for patients with advanced PD-L1–positive cervical cancer whose disease progressed following first-line chemotherapy.

The modest activity of single-agent CPIs in patients with cervical cancer led to studies evaluating multiple combinations, including of PD-1 and VEGF (156). KEYNOTE-826 and the BEATcc studies are ongoing Phase III studies evaluating the combination of PD-(L)1 and VEGF antibodies on a chemotherapy backbone (167, 168).

#### Ovarian Cancer

Epithelial ovarian cancer (EOC) accounts for over 95% of cases of ovarian cancer (169). Chemotherapy combined with bevacizumab is a standard of care for patients with newly diagnosed or recurrent disease (170). Despite EOC having favorable immune characteristics (high levels of TILs, neoantigens, and PD-L1 expression), the activity of PD-1/PD-L1 antibodies in EOC is modest, indicating the need for combination approaches to enhance antitumor immunity (171).

Given the pathogenic role of angiogenesis and the clinical utility of bevacizumab in EOC, a rationale exists for combined PD-(L)1–VEGF blockade. Atezolizumab plus bevacizumab was studied in a single-arm Phase II trial of 38 women with relapsed EOC: 18 with platinum-resistant and 20 with platinum-sensitive disease (172). The overall confirmed ORR was 29%; ORR was 40% and 17% in platinum-sensitive and platinum-resistant patients, respectively. A single-arm Phase II trial reported a response rate of 40% in platinum-resistant patients with recurrent EOC with the combination of bevacizumab, pembrolizumab, and metronomic oral cyclophosphamide (173).

Phase III studies in patients with advanced EOC are ongoing. The ATALANTE trial is comparing the combinations of chemotherapy with bevacizumab and atezolizumab vs. chemotherapy and bevacizumab alone in platinum-sensitive relapsed disease, while IMagyn050 is exploring this strategy in first-line treatment of newly diagnosed disease (**Table 3**). Alternative combinations that build on CPI/VEGF blockade are also under study. The emergence of poly (ADP-ribose) polymerase (PARP) inhibition as a treatment for EOC has provided justification to explore triplet therapy with a CPI, anti-VEGF, and PARP inhibitor (174). A Phase III study of durvalumab, bevacizumab, and olaparib is ongoing (175).

### Hepatocellular Carcinoma

HCC is highly angiogenic, as evidenced by hypervascularity, marked vascular abnormalities, and frequent overexpression of angiogenic factors such as VEGF (176, 177). Reflecting this vascular biology, most treatments currently approved for advanced HCC are either oral agents that inhibit angiogenic kinases or monoclonal antibodies against VEGFR (176, 178, 179). Despite initially appearing to offer a marked therapeutic advance, antiangiogenic drugs have shown modest survival improvements and low response rates, resulting in limited clinical benefit (176).

HCC is associated with inflammation and a suppressed immune environment, making CIT approaches a rational therapeutic approach (180–183). Encouraging early clinical data from two single-arm trials of pembrolizumab and nivolumab in advanced HCC formed the basis for the accelerated approval by the FDA (184, 185). In patients previously treated with sorafenib, response rates with nivolumab and pembrolizumab were 20% and 17%, respectively (184, 185). Despite these encouraging preliminary data, randomized Phase III trials of anti–PD-1 monotherapy in either first-line (nivolumab vs. sorafenib) or second-line (pembrolizumab vs. placebo) settings did not demonstrate statistically significant improvements in OS (186, 187). CheckMate 459, a Phase III study evaluating nivolumab vs. sorafenib as a first-line treatment in patients with unresectable HCC, did not achieve significance for its primary endpoint of OS (HR, 0.85; *P =* 0.075). Likewise, KEYNOTE-240, a Phase III trial evaluating pembrolizumab in patients who had previously received systemic therapy, did not achieve the prespecified OS boundary for statistical significance (HR, 0.781; *P =* 0.0238). These data likely highlight the strongly immunosuppressive nature of HCC and indicate the critical need for combination strategies to address additional immune defects beyond PD-(L)1.

Co-targeting the PD-(L)1 and VEGF signaling axes is the most extensively studied combination approach for advanced HCC (188, 189). Results from single-arm studies showed that combinations of VEGF and PD-(L)1 inhibitors were associated with a manageable safety profile and promising antitumor activity, with ORRs of 11% to 50% (190–195).

Of these combinations, atezolizumab and bevacizumab has been the most widely studied to date in HCC. A confirmed ORR of 36%—including a complete response rate of 12%—was reported in patients with unresectable HCC treated with atezolizumab and bevacizumab (106). Subsequently, combined atezolizumab and bevacizumab was evaluated in patients with unresectable HCC in two randomized studies, the results of which have been recently reported (106, 107, 196). These studies were designed to determine: (1) does bevacizumab augment the efficacy of atezolizumab treatment; and (2) is atezolizumab in combination with bevacizumab more effective than sorafenib for unresectable HCC?

In Arm F of study GO30140, 119 patients with unresectable HCC were randomly assigned 1:1 to receive either atezolizumab alone or atezolizumab plus bevacizumab (106). The primary endpoint was PFS assessed by an independent review facility. A statistically and clinically significant improvement in PFS was observed with the combination vs. atezolizumab monotherapy (HR, 0.55; *P =* 0.0108), with a median of 5.6 months vs. 3.4 months, respectively. Surprisingly, ORR was not markedly higher in the combination arm than in the atezolizumab arm (20% vs. 17%); however, the disease control rate was improved in favor of the combination (67% vs. 49%) (106). These data indicate that anti-VEGF treatment significantly enhances the efficacy of PD-L1 inhibition and a combination of PD-L1 and VEGF blockade is likely required to augment anticancer immunity in patients with unresectable HCC.

These encouraging findings led to the several randomized Phase III trials comparing these combination regimens with current standards of care (**Tables 3** and **4**).

IMbrave150 was a randomized Phase III study in which 501 patients with unresectable HCC were randomly assigned, in a 2:1 ratio, to receive either atezolizumab plus bevacizumab or sorafenib (a standard first-line anti-VEGF treatment). Co-primary endpoints were PFS (by blinded independent review) and OS. The results of IMbrave150 showed that atezolizumab plus bevacizumab resulted in a significant improvement in both PFS (HR, 0.59; *P* < 0.0001) and OS (HR, 0.58; *P* = 0.0006) compared with sorafenib (107). Further emphasizing the superior clinical benefit of combination therapy, ORR by central assessment more than doubled with atezolizumab plus bevacizumab compared with sorafenib alone (27% vs. 12%, *P* < 0.0001). Importantly, analysis of patient-reported outcomes showed significant and consistent benefits in quality of life, functioning, and key symptoms with atezolizumab plus bevacizumab compared to sorafenib, further supporting the overall clinical benefit of this combination (197). Based on the results of IMbrave150, the combination of atezolizumab and bevacizumab was recently approved by the FDA for the treatment of unresectable HCC, and it is expected that this combination will become a new standard of care (198, 199).

The clinical benefit of combined anti-VEGFR TKIs and CPIs in HCC remains to be validated in randomized studies. Several Phase III studies are currently ongoing assessing the combination of PD-(L)1 antibodies and VEGFR TKIs, including pembrolizumab combined with lenvatinib, atezolizumab plus cabozantinib, and camrelizumab (SHR-1210) with apatinib in advanced HCC, the results of which will clarify the utility of antiangiogenic TKIs as immunomodulators in conjunction with CPIs (**Table 3**) (190).

### Liver Metastases

The liver is a common metastatic site for most gastrointestinal (GI) cancers as well as for some non-GI tumors, such as lung cancer, renal cancer, breast cancer, and melanoma (200, 201). The presence of liver metastases is a negative prognostic factor in patients with lung and other cancers treated with CPIs (8, 202–204).

Differential organ responses in the liver vs. other anatomic sites have been recently reported in subgroup analyses from Phase III trials and retrospective series. Studies of CPIs have shown minimal therapeutic benefit as single agents or in combination with chemotherapy in patients with NSCLC and baseline liver metastases (29, 203, 205). In a subgroup of patients with metastatic melanoma treated with pembrolizumab as part of the KEYNOTE-001 trial, lung metastases were found to have the highest rate of complete response (42%), followed by peritoneal (37%) and liver (24%) metastatic lesions (206). In 90 patients with advanced malignancies (mostly melanoma and GI tumors) treated with CPIs in Phase I trials, the presence of liver metastasis was significantly associated with shorter OS, PFS, and lower rate of clinical benefit (204). In a retrospective review of 75 patients with advanced HCC, ORRs in the liver, lung, lymph node, and other intra-abdominal metastases were 22%, 41%, 26%, and 39%, respectively (207). Together, these clinical data suggest that hepatic metastases may be less responsive to CPIs than extrahepatic lesions.

One possible explanation for these clinical findings could be that secondary liver tumors harbor a more suppressive TME than primary anatomic sites. Consistent with this idea, results of a longitudinal analysis of metastases from a single patient with advanced ovarian cancer showed that each tumor deposit harbored divergent tumor genetics and distinct TMEs that evolved over time (208). Interestingly, progressing metastases were characterized by an immune cell excluded phenotype, whereas shrinking and stable metastases were well infiltrated by effector T cells and exhibited oligoclonal expansion of specific T-cell subsets (208). The presence of liver metastases from CPI-treated patients with NSCLC or melanoma was associated with abrogated CD8^+^ T-cell infiltration (202). Differential hepatic CPI responses also conceptually align with the idea of organ-specific immunoregulation—or “immunostat”—which hypothesizes that tissue-specific factors within the liver can modulate the sensitivity of metastatic deposits derived from other sites to CPIs (102). This may, in part, be due to the unique immune biology of the liver which acts to promote tolerance and an immunosuppressive TME (183, 209, 210).

Recent clinical data from randomized trials support the notion that CPIs combined with anti-VEGF agents could augment response to CPI treatment in patients with secondary liver tumors. In a pre-specified analysis from IMpower150, atezolizumab combined with bevacizumab and chemotherapy significantly improved OS and PFS in a subgroup of NSCLC patients with liver metastases (**Figure 3**). Conversely, neither atezolizumab plus chemotherapy or bevacizumab combined with chemotherapy did not prolong survival or PFS in patients with liver metastases (154). This indicates that the dual targeting of PD-L1 and VEGF may be needed to induce clinically meaningful antitumor immunity in NSCLC patients with liver metastases. Collectively, these clinical data suggest that the combination of bevacizumab and atezolizumab in patients with primary or secondary liver cancers may thwart the induction of immunosuppressive immune cell types (e.g., MDSCs, Tregs, and TAMs) that are induced by tumor hypoxia, VEGF overexpression, or increased hepatic angiogenesis (50).

## Challenges and Future Opportunities: Where Do We Go from Here?

A wealth of preclinical and clinical data supports the critical role that angiogenesis plays in modulating immunity in the TME. Randomized phase III studies have now shown that treatment combining antiangiogenics with a PD-(L)1 antibody significantly increased survival compared to standard-of-care treatment in RCC, NSCLC, and HCC. Results from ongoing randomized studies will further clarify the clinical benefit of this treatment approach in other types of cancer.

So far, anti-VEGF plus CPI combinations appear particularly effective in tumors for which antiangiogenesis and PD-(L)1 blockade are effective as individual monotherapies. It therefore remains to be seen whether CPI/VEGF combinations are efficacious in diseases such as ovarian cancer and MSS colorectal cancer that are angiogenic but often lack markers of preexisting immunity and respond poorly to PD-(L)1 antibody monotherapy. If randomized studies of CPI/VEGF-inhibitor combinations in poorly immunogenic cancers are positive, we will have compelling clinical evidence that switching or reprograming a cold TME to one that is immunogenic is a realistic clinical proposition.

Combinations of antiangiogenic agents in combination with CPIs have been studied with either anti-VEGF antibodies or TKIs; however, it is not clear whether the efficacy of these two approaches with respect to augmenting antitumor immunity are comparable. A key question is, how important is the choice of antiangiogenic when it is combined with a CPI? Antiangiogenic TKIs inhibit a broad spectrum of tyrosine kinases and do not only inhibit proangiogenic signaling pathways, whereas antibodies are directed against either VEGF-A or VEGFR2. The contribution of non-VEGF angiogenic kinases or other oncogenic pathways to TME immunomodulation remains to be delineated. Relatedly, antiangiogenic TKIs with different target inhibition profiles may possess differential immunomodulatory capacities. On one hand, TKIs may leverage additional immune-promoting mechanisms *via* a broader biological activity against angiogenesis; on the other hand, differences between TKIs and VEGF antibodies in safety profile and toxicity burden may be important determinants of clinical benefit, treatment duration, or combinability with other treatments. Identification of the optimal dose of antiangiogenic agents for immune modulation is critical for success in the clinic. A recent systematic review of the immune effects of antiangiogenic TKI drugs in preclinical models concluded that low doses were immunostimulatory, whereas higher doses were immunosuppressive (211). This aligns with other preclinical data in tumor models that suggest that antiangiogenic therapies that are high dose, long term, or both can cause excessive vessel pruning and increased immunosuppression (13). The clinical significance of anti-VEGF dose (higher vs. lower) remains to be determined. Notably, data from completed Phase III studies in RCC, NSCLC, and HCC all used standard FDA-approved doses of antiangiogenic agents. The optimal duration of treatment and sequencing of drugs is also an important consideration that will require evaluation in well-controlled clinical studies.

In addition to the choice of anti-VEGF agent, the choice of CPI may also be relevant. No direct head-to-head clinical studies contrasting PD-1 and PD-L1 antibodies are available. Indirect data from systematic reviews or meta-analyses, mostly in NSCLC, are inconsistent with those from some studies showing no difference in efficacy between PD-1 or PD-L1 antibodies and from others indicating improved survival in favor of PD-1 inhibitors (212–214). Differential clinical efficacy of receptor- vs. ligand-based PD-1 blockade may be partially a function the tumor type being treated (214). Recent *in vitro* studies suggest that differences between receptor- vs. ligand-based antagonism may exist and have implications for combination treatments. In a study using a functional T-cell *in vitro* assay, PD-L1 antibodies were found to be more effective than PD-1 antibodies in inhibiting PD-1 signaling (215). A study using *in vivo* murine breast and colon cancer models showed that anti–PD-L1 (but not anti–PD-1) monotherapy was able to deplete CD80 ligand expression on tumor-infiltrating antigen-presenting cells, thereby inhibiting CTLA-4 axis through a Treg-dependent mechanism (216). The role of Tregs in this model is intriguing when considering anti–PD-(L1)/VEGF combinations given the role that Tregs play in VEGF-mediated immunosuppression. The clinical implications of these basic research data remain to be seen, and the results of ongoing trials with different combinations of PD-1/PD-L1 antibodies and anti-VEGF agents will be informative.

The encouraging results from Phase III trials of CPIs combined with anti-VEGF agents and their adoption as standards of care for patients with advanced disease motivates consideration of this approach in earlier, potentially curative, treatment settings. Anti–PD-(L)1 antibodies are currently approved as adjuvant treatment following resection in melanoma and following chemoradiation in NSCLC (217–219). Two Phase III studies are currently ongoing to evaluate anti–PD-L1 antibodies combined with bevacizumab in patients with HCC at high risk of tumor recurrence following potentially curative liver resection or tumor ablation (220, 221). These adjuvant studies are predicated on the hypothesis that dual PD-L1/VEGF blockade may reduce HCC recurrence by creating a more immune-favorable TME (221). PD-L1/VEGF blockade is also under study in a Phase III trial in combination with transarterial chemoembolization in patients with unresectable liver-confined HCC. This combination is based on the potential to amplify antitumor immune mechanisms induced by locoregional treatment (222).

Antiangiogenics combined with PD-1/PD-L1 antibodies are now standard-of-care frontline treatments for NSCLC, RCC, endometrial cancer, and HCC. These successes may represent the tip of the iceberg in efficacious combinations of CPIs and TME-modulating agents. At present, 76% of the almost 3000 PD-1/PD-L1 antibody clinical trials are testing combination regimens (7). This will likely result in continued rapid evolution of cancer treatment algorithms, potentially adding complexity to the processes of personalization and determining the right treatment approach for a patient’s specific disease. In the era of combination CIT and modulation of specific facets of the TME, biomarker development is challenging. Unlike molecularly targeted drugs for which diagnostic biomarkers are typically a specific genetic aberration defined as a binary (yes or no) assay, CIT biomarkers are often continuous variables that have a gradation of association with clinical endpoints (12). Data so far suggest that established CIT biomarkers such as PD-L1 expression or TMB have limited utility for dual CPI/VEGF blockade. Molecular profiling of the TME may represent a useful approach to identify patients with TME immune defects mediated by VEGF (223). For example, exploratory data from randomized studies in patients with RCC or HCC harboring a myeloid gene signature suggest that the use of bevacizumab with atezolizumab may be beneficial. Further translational studies that include either paired serial biopsies or neoadjuvant approaches will be needed to identify mechanisms of response and resistance to CIT treatment. The learnings from these types of trials will enable the rational development of next-generation combinations in which drugs targeting specific immune suppressive mechanisms in the TME are added to a PD-(L1)/VEGF backbone. The search for predictive biomarkers in order to better select patients for CIT treatment is ongoing, but as yet no biomarkers have been validated for use in clinical practice.

## Conclusion

Combined blockade of PD-(L)1 and VEGF pathways represents a significant therapeutic advance in cancer treatment. The immunomodulatory role of VEGF, now well described by data from preclinical and translational studies as well as randomized clinical trials, provides a compelling reason to continue the study of anti-VEGF and immune checkpoint therapies across the cancer spectrum. Ongoing trials will continue to discern the immunological mechanisms underpinning this treatment approach and will further delineate the clinical benefit of this approach for the treatment of cancer.

## Author Contributions

SH wrote the first draft of the manuscript. All authors contributed to the article and approved the submitted version.

## Funding

Professional editorial assistance for this manuscript was provided by Health Interactions, Inc. and funded by F. Hoffmann-La Roche, Ltd. The funder bodies were not involved in the study design, collection, analysis, interpretation of data, the writing of this article or the decision to submit it for publication.

## Conflict of Interest

SH and YW are fulltime employees of Roche/Genentech who hold Roche shares and stock options. AZ reports consulting and advisory roles for Lilly, Bayer, Merck, Sanofi, Eisai, Exelixis, and Roche.

## Abbreviations

5-FU, fluorouracil; ABCP, atezolizumab plus bevacizumab plus carboplatin plus paclitaxel; ALK, ALK receptor tyrosine kinase; BCP, bevacizumab plus carboplatin plus paclitaxel; CIC, cancer-immunity cycle; CIT, cancer immunotherapy; CPI, checkpoint inhibitors; CTLA-4, cytotoxic T-lymphocyte-associated protein 4; DCs, dendritic cells; dMMR, deficient DNA mismatch repair pathways; EGFR, epidermal growth factor receptor; EOC, epithelial ovarian cancer; FDA, US Food and Drug Administration; FOLFOX, leucovorin calcium, fluorouracil, and oxaliplatin; HCC, hepatocellular carcinoma; ICIs, immune checkpoint inhibitors; IFN, interferon; ITT, intention-to-treat; mCRC, metastatic colorectal cancer; MDSCs, myeloid-derived suppressor cells; MEK, mitogen-activated protein kinase kinase; MHC, major histocompatibility complex; MSI-H, high microsatellite instability; MSS, microsatellite stable; NSCLC, non-small cell lung cancer; ORR, objective response rate; OS, overall survival; PD-1, programed cell death protein 1; PD-L1, programed cell death ligand 1; PFS, progression-free survival; pMMR, proficient DNA mismatch repair pathways; RCC, renal cell carcinoma; TAMs, tumor-associated macrophages; TILs, tumor-infiltrating lymphocytes; TKI, tyrosine kinase inhibitor; TME, tumor microenvironment; Treg, regulatory T cells; VEGF, vascular endothelial growth factor; VEGFR, vascular endothelial growth factor receptor.
