# Supplementary material for: Augmenting Anticancer Immunity Through Combined Targeting of Angiogenic and PD-1/PD-L1 Pathways: Challenges and Opportunities
Source: Front Immunol. 2020 Nov 5;11:598877. doi: 10.3389/fimmu.2020.598877 (PMC7674951; doi:10.3389/fimmu.2020.598877)
Supplement: Supplementary file 1 [file Table_1.docx]

**Supplement**

|  | Cancer Indication^a^ | | | | | | | | | | | | | | | | | | |
| --- | --- | --- | --- | --- | --- | --- | --- | --- | --- | --- | --- | --- | --- | --- | --- | --- | --- | --- | --- |
|  | **Melanoma** | **NSCLC** | **Mesothelioma** | **RCC** | **Hodgkin Lymphoma** | **SCCHN** | **Merkel Cell Carcinoma** | **MSI-H/dMMR Cancers** | **Colorectal Cancer (MSI/dMMR)** | **Gastric Cancer** | **HCC** | **Cervical Cancer** | **PMBCL** | **SCLC** | **CSCC** | **Bladder Cancer** | **Breast Cancer** | **Endometrial Cancer** | **Esophageal Cancer** |
| Monotherapy | US  EU  CN  JP | US  EU  CN  JP | JP | US  EU  JP | US  EU  CN  JP | US  EU | US  EU  JP | US  JP | US | US  JP | US | US | US | US | US  EU | US  EU  JP | – | – | US  JP |
| With αCTLA-4 | US  EU | – | – | US  EU | – | – | – | – | US | – | US | – |  | – | – | – | – | – | – |
| With Chemo | – | US  EU  CN  JP | – | – | – | US  EU | – | – | – | – | – | – |  | US  JP | – | – | US | – | – |
| With αVEGF | – | US  EU | – | US  EU  JP | – | – | – | – | – | – | US | – |  | – | – | – | – | US | – |

**Supplemental Table 1. Approved cancer indications for PD-1/PD-L1 antibodies in major countries or regions^b^**

αCTLA-4, anti–CTLA-4 monoclonal antibody; αVEGF, anti-VEGF drug; CN, China; CSCC, cutaneous squamous-cell carcinoma; dMMR, deficient DNA mismatch repair; EU, Europe; HCC, hepatocellular carcinoma; JP, Japan; –, not approved; MSI-H, microsatellite instability-high; NSCLC, non-small cell lung cancer; PMBCL, primary mediastinal B-cell lymphoma; RCC, renal cell carcinoma; SCCHN, squamous cell carcinoma of the head and neck; SCLC, small-cell lung cancer; US, United States of America.

^a^ Includes both full and accelerated health authority approvals.

^b^Approved anti–PD-1/PD-L1 monoclonal antibodies: atezolizumab (Roche), avelumab (Pfizer/Merck KGaA), camrelizumab (Jiangsu HengRui Medicine), cemiplimab (Regeneron), duvalumab (AstraZeneca), nivolumab (Bristol-Myers Squibb), pembrolizumab (Merck), sintilimab (Innovent Biologics and Eli Lilly), tislelizumab (Beigene), toripalimab (Junshi Biosciences).

Source: <https://www.cancerresearch.org/scientists/immuno-oncology-landscape/pd-1-pd-l1-landscape> (date accessed, March 11, 2020)

| Drug | Target(s) | FDA-Approved Indications^a^ |
| --- | --- | --- |
| Bevacizumab | VEGF-A | - First- or second-line mCRC (with fluorouracil-based chemotherapy) - Second-line mCRC following progression on a first-line bevacizumab-containing regimen (with fluoropyrimidine-irinotecan- or fluoropyrimidine-oxaliplatin-based chemotherapy) - First-line unresectable, locally advanced, recurrent, or metastatic NSCLC (with carboplatin and paclitaxel) - Recurrent glioblastoma in adults - Metastatic renal cell carcinoma (with interferon-α) - Persistent, recurrent, or metastatic cervical cancer (with paclitaxel and cisplatin, or paclitaxel and topotecan) - Epithelial ovarian, fallopian tube, or primary peritoneal cancer:   - with carboplatin and paclitaxel for stage III or IV disease following initial surgical resection   - with paclitaxel, pegylated liposomal doxorubicin, or topotecan for platinum-resistant recurrent disease who received no more than 2 prior chemotherapy regimens   - with carboplatin and paclitaxel or carboplatin and gemcitabine for platinum-sensitive recurrent disease |
| Ziv-aflibercept | VEGF-A,  VEGF-B, and PlGF | - mCRC (with FOLFIRI) |
| Ramucirumab | VEGFR2 | - Second-line advanced or metastatic gastric or gastro-esophageal junction adenocarcinoma (as a single agent or in combination with paclitaxel) - Second-line metastatic NSCLC (with docetaxel) - Second-line mCRC (with FOLFIRI) - Second-line HCC with an α-fetoprotein of ≥400 ng/mL |
| Cabozantinib | MET, VEGFR2, and TIE2 TKI | - Advanced RCC (after VEGFR TKI treatment) - Second-line HCC (after sorafenib) |
| Regorafenib | VEGFR2/3, RET, KIT, TIE2, PDGFR, and RAF | - Locally advanced, unresectable or metastatic GIST (after imatinib and sunitinib) - Chemorefractory mCRC - HCC (after sorafenib) |
| Sorafenib | VEGFR1-3, PDGFRα/β, FGFR1-4, KIT, RET, FLT3, CRAF, BRAF, and TIE2 | - Unresectable HCC - Advanced RCC - Locally recurrent or metastatic, progressive, DTC refractory to radioactive iodine treatment |
| Axitinib | VEGFR1-3 and PDGFRβ TKI | - Second-line advanced RCC |
| Lenvatinib | VEGFR1-3, PDGFRα, and FGFR1-4 | - Recurrent or metastatic radioactive iodine-refractory DTC - Advanced RCC (with everolimus) |
| Pazopanib | KIT, FGFR1/2, PDGFRβ, and VEGFR1-3 | - Advanced soft-tissue sarcoma (after chemotherapy) - Advanced RCC |
| Sunitinib | VEGFR1-3, PDGFRα/β, KIT, RET, FLT3, and G-CSF-R | - Advanced clear-cell RCC (with IFN-ɑ) - GIST (after failure of imatinib) - Advanced PNET - High-risk clear-cell RCC (after nephrectomy) |
| Vandetanib | VEGFR1-3, EGFR, RET, and PDGFRα/β | - Unresectable, locally advanced, or metastatic MTC |

**Supplementary Table 2. FDA-approved agents targeting the VEGF axis**

mCRC, metastatic colorectal cancer; DTC, differentiated thyroid carcinoma; FDA, US Food and Drug Administration; FOLFIRI, leucovorin, fluorouracil, and irinotecan; G-CSF-R, granulocyte colony-stimulating factor receptor; GIST, gastrointestinal stromal tumor; HCC, hepatocellular carcinoma; IFN-ɑ, interferon ɑ; MTC, medullary thyroid carcinoma; NSCLC, non-small cell lung cancer; PDGFR, platelet-derived growth factor receptor; PFS, progression-free survival; PlGF, placenta growth factor; PNET, pancreatic neuroendocrine tumor; RCC, renal cell carcinoma; TKI, tyrosine kinase inhibitor; TIE2, angiopoietin 1 receptor; VEGF, vascular endothelial growth factor; VEGFR, vascular endothelial growth factor receptor.

^a^ Based on US prescribing information (accessed March 8, 2020).
